# Small Bowel Cancer: Implications of Preoperative Work‐Up on Surgical Strategy

**DOI:** 10.1002/wjs.70547

**Published:** 2026-09-03

**Authors:** Erik Kjæstad, Espen Thiis‐Evensen, Anita Sveen, Arne Olav Bakka, Johannes Kurt Schultz

**Affiliations:** ^1^ Dept. Gastrointestinal Surgery Akershus University Hospital Lørenskog Norway; ^2^ Institute of Clinical Medicine University of Oslo Oslo Norway; ^3^ Dept. Transplantation Medicine Centre for Neuroendocrine Tumors Oslo University Hospital Oslo Norway; ^4^ Dept. Molecular Oncology Institute for Cancer Research Oslo University Hospital Oslo Norway

**Keywords:** diagnostics, small bowel cancer, small intestinal adenocarcinoma, small intestinal neuroendocrine tumors, subgroups, surgery

## Abstract

**Background:**

Small bowel cancers comprise a heterogeneous group of malignancies, making diagnosis and treatment challenging. This study aimed to evaluate the ability of preoperative diagnostic work‐up to predict clinical and histopathological findings, facilitate subgroup identification, and support tailored, individualized surgical strategies.

**Methods:**

This retrospective single‐center analysis included all patients operated 2015–2024 for suspected small bowel cancer at a tertiary referral center. Preoperative assessments—including diagnostic evaluation of tumor type, disease extent, and anatomical localization—were compared to intraoperative findings and final histopathological results.

**Results:**

Of 187 operations, 59 (32%) were right colectomies, 50 (27%) robotic/laparoscopic procedures and 36 (19%) emergencies. Preoperative symptoms were present in 156 (83%) patients. CT scans were performed in 184 (98%), somatostatin receptor imaging (SRI) in 120 (64%), endoscopy in 92 (49%), and biopsy in 74 (40%). Small intestinal neuroendocrine tumors (siNET) were found in 108 (58%) patients, carcinomas in 24 (13%), metastatic tumors in 17 (9%), gastrointestinal stromal tumors in 13 (7%), benign lesions in 13 (7%), lymphomas in 8 (4%), and desmoid tumors in 4 (2%). The preoperative tentative tumor entity was correct in 118 (63%). CT sensitivities for mesenteric and peritoneal metastases were 65% and 9%, respectively. SRI was positive in 83/92 siNET and 6/28 non‐NET. Mesenteric metastases were found in 99 (95%) siNET patients. SRI failed to identify mesenteric dissemination in 24 (27%) siNET patients. Among patients with siNET, 18 (17%) had liver metastases and 18 (17%) had peritoneal metastases. SiNET multifocality was associated with distant metastatic status (*p* < 0.001). Pathologists found 313 luminal siNET, 17 tumors were missed by intraoperative palpation, 51 by SRI and 156 by CT. All siNET were in the ileum. Terminal compared to middle/upper ileum siNET were more often solitary (39/42 [93%] vs. 31/60 [52%]; *p* < 0.001) and had less often advanced mesenteric disease (N2) (9/44 [20%] vs. 31/58 [53%]; *p* < 0.001).

**Conclusions:**

Preoperative work‐up of small bowel cancer is often inaccurate, particularly when it comes to identification of bowel tumors and metastases. Intraoperative palpation of the bowel and its mesentery and inspection of the peritoneum remain essential to facilitate an adaptive surgical strategy.

## Graphical abstract

Small bowel cancers comprise a heterogeneous group of malignancies, making diagnosis and treatment challenging. This study aimed to evaluate the ability of preoperative diagnostic work‐up to predict clinical and histopathological findings, facilitate subgroup identification, and support tailored, individualized surgical strategies. Preoperative work‐up of small bowel cancer is often inaccurate, particularly when it comes to identification of bowel tumors and metastases. Intraoperative palpation of the bowel and its mesentery and inspection of the peritoneum remain essential to facilitate an adaptive surgical strategy.

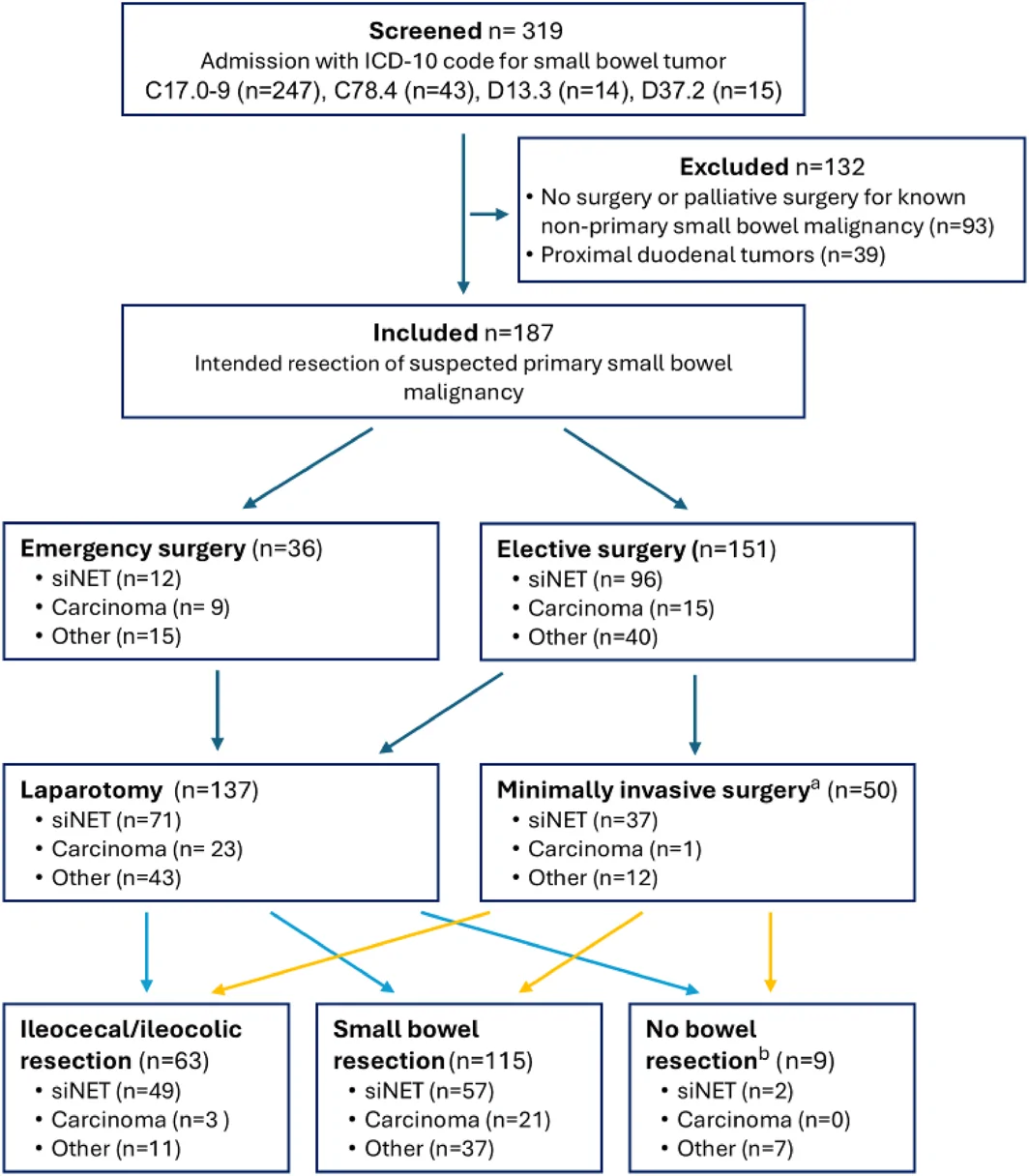

## 1 Introduction

Small bowel malignancies are rare but encompass a large variety of tumor entities [1], posing challenges in the establishment of standardized diagnostic and therapeutic approaches. In Western countries, small intestinal neuroendocrine tumors (siNET) have surpassed adenocarcinomas as the most frequent entity [2], followed by lymphomas, gastrointestinal stromal tumors, metastases and sarcomas [3].

Radiology and nuclear medicine imaging are used in the work‐up of these malignancies. Contrast enhanced computed tomography (CT) is the most used modality [4, 5, 6]. For siNET, CT‐sensitivity as low as 35%–36% has been reported [7, 8] increasing up to 90%–95% when performed as CT angiography or enterography series [8, 9]. Contrast enhanced magnetic resonance imaging (MRI), ^18^F‐fluorodeoxyglucose positron emission tomography (FDG‐PET) and somatostatin receptor imaging (SRI) are often helpful. SRI is effective in diagnosing well‐differentiated siNET [10] and can be scintigraphy‐based or PET‐based [11, 12]. Still, there are difficulties in detecting small and multiple tumors [13] and the extent of mesenteric disease can be masked by fibrosis and desmoplasia or dominating tumor uptakes [7, 14]. Although enteroscopy allows biopsies, it is technically challenging, time consuming and not all parts of the small bowel can be visualized [15]. Video capsule enteroscopy can visualize most of the small bowel, but without the possibility of biopsies and with the risk of capsule entrapment [16, 17]. Despite optimization of these modalities, the precise etiology of a small bowel tumor may remain unknown prior to surgery.

Treatment recommendations in multi‐disciplinary board meetings and shared decision making prior to surgery rely on preoperative work‐up quality, as does the evolvement of a detailed tentative surgical strategy. Modern small bowel cancer surgery includes minimally invasive procedures and extended lymphadenectomy with bowel‐sparing techniques. Clearance of mesenteric disease is widely recommended, to achieve symptom control, correct staging and possibly improved survival, and serves as an indirect measure of surgical quality [17, 18, 19, 20, 21, 22]. Although central lymphadenectomy has been described for siNET [23, 24, 25], there is no consensus on the extent of lymphadenectomy [26]. Multifocality is common in siNET [27], and it has been shown that even small primary tumors frequently metastasize [28]. Many experts recommend resecting primary siNET also in patients with distant metastatic disease, both for symptom control and to improve survival outcomes [29, 30]. Intraoperative findings of more extensive disease, including lesions in previously unknown locations, may necessitate deviations from the planned surgical strategy.

The aim of this study is to assess the diagnostic accuracy of contemporary evaluation methods for small bowel cancer, characterize its heterogeneity, and identify patient subgroups that may benefit from distinct treatment strategies.

## 2 Methods

### 2.1 Inclusion

This single‐center retrospective cohort study included patients operated for suspected or biopsy verified small bowel cancer at Dept Gastrointestinal Surgery, Akershus University Hospital, Norway, during the 10‐year period 01/01/2015–12/31/2024. A board‐approved quality control database was checked against the electronic journal system by searching ICD‐10 [31] diagnoses of small bowel tumors of malignant, benign or unknown origin, as well as metastases to the small bowel. Surgical records in the resulting journals were analyzed. Distal duodenal and duodenojejunal flexure cancers suited for pancreas‐sparing duodenal resections were included and proximal duodenal cancers excluded. Patients operated with curative, symptom relief and debulking intentions were included. The principles of the STROBE guidelines (Strengthening the Reporting of Observational Studies in Epidemiology) [32] were followed.

### 2.2 Data Retrieval and Variables

Baseline characteristics, disease presentation, and findings of preoperative work‐up performed within 4 months prior to surgery were recorded, as well as the tentative preoperative diagnosis, tumor stage, surgical treatment, intraoperative and histopathological findings. A new classification of small bowel tumors was applied, dividing the bowel into five sectors according to primary tumor and mesenteric disease location (feeding artery ligation). Surgical charts were reviewed to identify resections and vessel ligations. Ileocolic artery (ICA) area is sector 1 (coecum and terminal ileum). Terminal superior mesenteric artery (SMA) area is sector 2a (middle ileum). Ileal arteries area is sector 2b (upper ileum). Jejunal arteries area is 2c (jejunum). Duodenojejunal (DJ) flexure supplied by the first jejunal artery is sector 3 (fourth part of the duodenum and first 10 cm of the jejunum). Ileal vessels are defined to origin from SMA caudally to the ICA origin, and jejunal arteries cranial to this. Multiple and multi‐sector resections were classified according to the location of the primary tumor and mesenteric mass. Non‐dominant secondary bowel tumors of the same entity were called synchronous tumors. It was recorded whether lymphadenectomies were minimal (close to the bowel, “D1”), intermediate (along vessel branches,” D2″) or extended (central vessel ligation, “D3”), similar to colorectal cancer surgery definitions [33]. Inspection of the entire peritoneal cavity and manual palpation of the small bowel and its mesentery was standard. In minimally invasive surgery, the bowel was exteriorized through a mini laparotomy.

### 2.3 Statistical Analysis

The IBM Statistics program SPSS 29.0 was used for comparative analyses of subgroups. The Fischer exact, Chi square, Mann‐Whitney U and Kruskal‐Wallis tests were used. Preoperative, intraoperative and histopathological evaluations were compared to analyze the accuracy of preoperative work‐up.

### 2.4 Ethics

The study was approved by the by the southeast Norwegian regional ethics committee (753582/REC southeast A) and active consent was waived. All patients still alive were informed about the use of their data and were given the possibility to opt out. A subgroup of patients was also included in the study “Safe extended (D3) mesenterectomy for small bowel tumors” (ClinicalTrials.gov ID NCT05670574).

## 3 Results

### 3.1 Study Population

Altogether, 187 patients were included (study flow chart in Figure 1). Baseline population characteristics and preoperative work‐up methods are presented in Table 1. Abdominal CT scans were performed in 184 (98%) patients. SRI was performed in 91/108 (84%) of siNET patients. The median number of investigations (radiological, nuclear medical, endoscopic) performed per patient within 4 months prior to surgery was 4 (range 0–10). Dominant symptoms at presentation were abdominal pain in 98 (52%), bowel obstruction in 41 (22%) and anemia in 37 (20%) patients. Emergency surgery was performed in 36 (19%) patients, due to bleeding in three (2%), bowel obstruction in 27 (14%), ischemia in one (1%) and perforation in five (3%).

**FIGURE 1:**
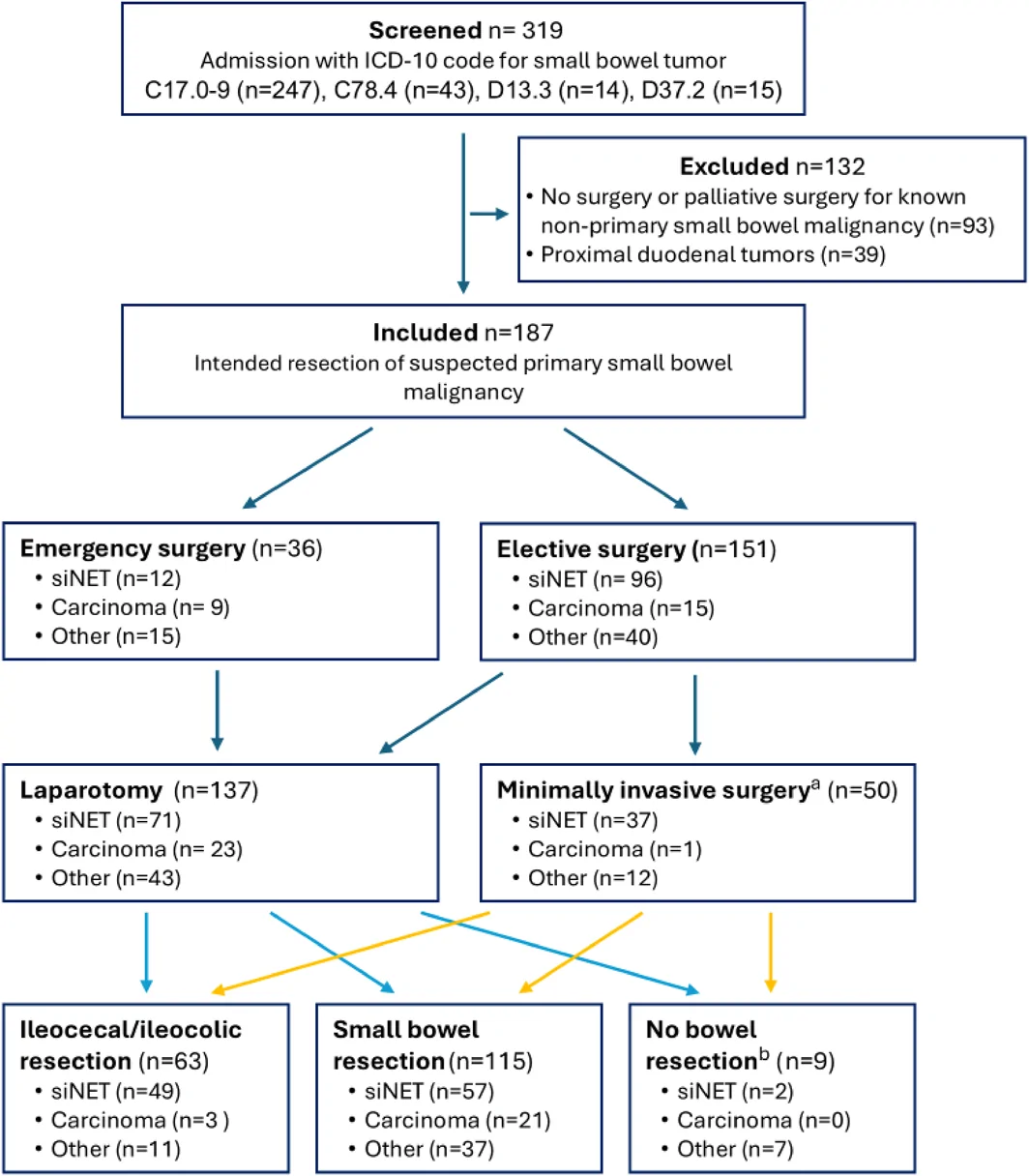
Study flow chart. ^a^ Conversion to laparotomy occurred in 10/50 minimally invasive procedures (20%). ^b^ Of nine cases of no bowel resection, six were mesenteric biopsies and three were exploratory laparotomies. ICD‐10: international statistical classification of diseases and related health problems, 10th revision. siNET: small intestinal neuroendocrine tumor.

**TABLE 1:** Baseline characteristics and preoperative work‐up.

	Total cohort	siNET	Carcinoma
	(*n* = 187)	(*n* = 108)	(*n* = 24)
Baseline characteristics			
Age: years—median (IQR)	65.0 (54.0–73.0)	62.0 (53.0–71.0)	72.0 (64.0–76.0)
Sex (female)	79 (42.2)	46 (42.6)	8 (33.3)
BMI: kg/m^2^—median (IQR)	25.6 (23.3–28.7)	26.6 (24.3–30.3)	25.1 (22.3–26.5)
ASA class			
1	16 (8.6)	9 (8.3)	0
2	107 (57.2)	62 (57.4)	14 (58.3)
3	62 (33.2)	36 (33.3)	9 (37.5)
4	2 (1.1)	1 (0.9)	1 (4.2)
Previous abdominal surgery	106 (56.7)	63/108 (58.3)	14 (58.3)
Preoperative investigation			
CT abdomen	184 (98.4)	106 (98.1)	24 (100.0)
More than one CT series	83 (44.4)	52 (48.1)	10 (41.7)
Proportion CT enterography protocols	44/271 (16.2)	27/160 (16.9)	4/34 (11.8)
MRI abdomen or small bowel protocol	15 (8.0)	13 (12.0)	0
Chest CT	143 (76.5)	89 (82.4)	18 (75.0)
Supplementary liver investigations (US, CT, MRI)	20 (10.7)	17 (15.7)	1 (4.2)
Somatostatin receptor imaging (SRI)	120 (64.2)	91 (84.3)	4 (16.7)
^99m^Tc‐tectrotyde scintigraphy	56 (29.9)	39 (36.1)	2 (8.3)
^68^Ga‐DOTATOC PET‐CT	69 (36.9)	57 (52.8)	2 (8.3)
^18^F‐fluorodeoxyglucose (FDG)‐PET	9 (4.8)	2 (1.9)	0
Endoscopy	92 (49.2)	50 (46.3)	16 (66.7)
Ileocoloscopy	59 (31.6)	43 (39.8)	5 (20.8)
Lower balloon enteroscopy	10 (5.3)	6 (5.6)	0
Gastroduodenoscopy	24 (12.8)	2 (1.9)	13 (54.2)
Upper balloon enteroscopy	6 (3.2)	0	2 (8.3)
Endoscopic ultrasound	2 (1.1)	0	0
Capsule enteroscopy	4 (2.1)	1 (0.9)	1 (4.2)
Tissue biopsy	74 (39.6)	46 (42.6)	14 (58.3)
Proportion taken from the small bowel	49/74 (66.2)	33/46 (71.7)	11/14 (78.6)

*Note:* Values are *n* (%) unless otherwise indicated.

Abbreviations: ASA = American Society of Anesthesiologist's Physical Status Classification System; BMI= Body Mass Index; CT = computed tomography; MRI = magnetic resonance imaging; SRI = somatostatin receptor imaging; siNET = small intestinal neuroendocrine tumor.

### 3.2 Surgery

Ileocecal or ileocolic resection was performed in 63 (34%) patients. Central (D3) lymphadenectomy was performed in 90 (48%) patients, as part of the study “Safe extended mesenterectomy for small bowel tumors” (*n* = 84) or due to suspicion of central malignant lymph nodes (*n* = 6). A D2 lymphadenectomy was performed in 71 (38%) and a D1 resection in 18 (10%) patients. Three (2%) patients had an explorative laparotomy and six (3%) a mesenteric resection; the reasons for omitting bowel resection and/or lymphadenectomy were intraoperatively benign findings, biopsied gastrointestinal stromal tumors, massive adhesions, or disseminated cancer. Most procedures were performed by the same team (one of two senior surgeons present at 142 [76%] of operations). Multi‐visceral resections were performed in 14 (8%) patients (five colon, two rectum, two liver, two ovaries, one pancreas, one breast, one paraaortic metastasis). Minimally invasive surgery was attempted in 50 (27%) patients, with 10 conversions. Multiple bowel resections (range 2–3) were done in five (5%) patients, all with siNET. For siNET, the median bowel resection length measured intraoperatively was 60 (range 10–160) centimeters and 31 (range 5–150) centimeters measured on the fixated specimen.

### 3.3 Surgical and Histopathological Findings

The most common histopathological entity was siNET, found in 108 (58%) patients, followed by 24 (13%) carcinomas, 16 (9%) metastases from other origin (including seven malignant melanomas), 13 (7%) gastrointestinal stromal tumors, eight (4%) lymphomas, four (2%) desmoid tumors, one (1%) leiomyosarcoma and 13 (7%) benign lesions. The indications for surgery in the benign group were suspicion of malignancy in CT (*n* = 8) or in PET scans (*n* = 4) or obstructive invagination (*n* = 1). Of the 108 siNET, 95 (88%) were grade I. Histopathological details of siNET and carcinomas are displayed in Supporting Information S1: Table 1. All tumors could be categorized by the new 5‐sector system of disease location. The distribution of tumors is illustrated in Figure 2. All siNET were in the ileum, with a median distance from the most proximal tumor to Treitz’ ligament of 282.5 cm (range 170–530). Two‐sector disease was found in seven (6%) siNET patients (type 1 plus 2a in four, and type 2a plus 2b in three). All these patients had their primary tumor and mesenteric deposits in sector 2a and were classified accordingly.

**FIGURE 2:**
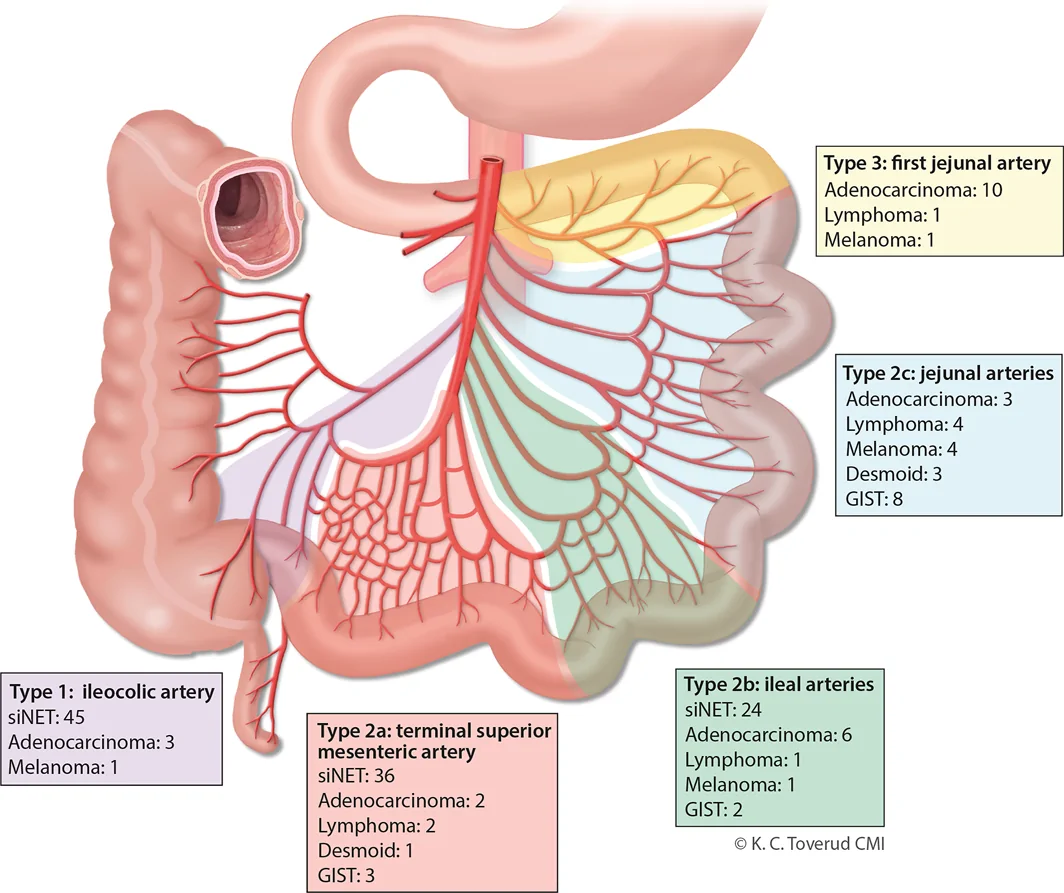
Distribution of different tumor entities. Sectors of small bowel according to feeding arteries of tumors and mesenteric masses. Type 1: Ileocecal resection or right colectomy. Type 2: Small bowel resection (2a: Middle ileum resection, 2b: Upper ileum resection, 2c: Jejunal resection). Type 3: Duodenojejunal resection. Adenocarcinoma dominated in the first parts and siNET in the last parts of the small bowel. All siNET were in the ileum. siNET: small intestine neuroendocrine tumor, GIST: gastrointestinal stromal tumor.

Altogether, 98 (91%) siNET patients had mesenteric metastases (N+) found at histopathology and 95 (88%) had ≥ 8 lymph nodes retrieved. An N2 status (≥ 12 mesenteric metastases and/or mesenteric mass diameter > 2 cm) was found in 42 (39%) patients (in 22 of these, the stage was based solely on the mesenteric mass size). Distant siNET metastases were identified either at staging or intraoperatively in 36 (33%) patients (18 liver, 18 peritoneal, six retroperitoneal, three mediastinal, two pancreas, two appendix, one female breast, one ovary, one bone metastasis). A free resection margin (R0) was achieved in 103 (95%) of siNET patients, while a histologically unfree margin (R1) and macroscopic residual tumor in the mesentery (R2) were found in three and two patients, respectively. Pathological examination identified a total of 313 luminal siNET in 105 patients, and 32 (31%) patients had tumor multiplicity (2–61 tumors). The number of bowel tumors was significantly associated with the presence of distant metastases (M+stage) (*p* < 0.001). Terminal compared to middle/upper ileum siNET were more often solitary (39/42 [93%] vs. 31/60 [52%]; *p* < 0.001) and less often N2 (9/45 [20%] vs. 31/58 [53%]; *p* < 0.001).

All 24 carcinomas except one were solitary and had a median tumor diameter of 37 mm (range 10–90). Carcinomas were found in all parts of the small bowel, with 10 (42%) at the duodenojejunal flexure. A T4‐status (perforation of visceral peritoneum or invasion of other organs) was found in 17 (71%) and mesenteric metastases (N+) in 17/23 (74%). There were three patients with liver metastases, five with carcinomatosis, two with lung metastases, and one with retroperitoneal metastases. Duodenojejunal carcinomas were N+ in 9/10 (90%) compared to 8/14 (57%) of distal carcinomas (*p* = 0.09). Distant metastases were found in 1/10 (10%) of DJ carcinomas versus 9/14 (64%) among more distal cancers (*p* < 0.01). All patients with DJ carcinoma had upper endoscopies and biopsies.

### 3.4 Diagnostic Accuracy

The preoperative work‐up predicted the correct histopathological tumor entity in 118 (63%) patients (Tables 2 and 3). Preoperative tissue biopsies correctly identified the entity in 57 (77%) of the 74 patients who underwent biopsy. CT correctly suggested the tumor type in 73/184 (40%) (five patients had known results of endoscopic biopsies prior to CT). CT sensitivity for mesenteric metastases was 65% (78/120). CT failed to detect 156/240 (65%) synchronous luminal siNET.

**TABLE 2:** Detection of malignant disease and tumor entity.

Investigation	Parameter identified	Total cohort (*n* = 187)	siNET (*n* = 108)	*Subgroup: Terminal ileal siNET(n = 45)*	Carcinoma (*n* = 24)	*Subgroup: Duodenojejunal carcinoma (n = 10)*
CT	Malignant disease	157/173 (90.8%)	98/106 (92.5%)		18/24 (75.0%)	
	Correct tumor entity	73/184 (39.7%)	66/106 (62.3%)		4/24 (16.7%)	
Endoscopy	Malignant disease	50/92 (54.3%)	31/50 (62.0%)	26/33 (78.8%)	10/16 (62.5%)	9/10 (90.0%)
Biopsy	Correct tumor entity	57/74 (77.0%)	38/46 (82.6%)	24/26 (92.3%)	10/14 (71.4%)	9/10 (90.0%)
FDG‐PET	Malignant disease	5/6 (83.3%)				
SRI	Correct tumor entity		82/91 (90.1%)			

*Note:* Results of preoperative investigations compared to final histopathological results. Number of patients in denominators.

**TABLE 3:** Detection of metastatic disease and staging accuracy.

Investigation	Parameter identified	Total cohort (*n* = 187)	siNET (*n* = 108)	Carcinoma (*n* = 24)
CT	Mesenteric metastases	78/120 (65.0%)	70/97 (72.2%)	3/17 (17.6%)
	Peritoneal metastases	2/22 (9.1%)	2/16 (12.5%)	0/5 (0.0%)
	Liver metastases^a^	17/22 (77.3%)	16/18 (88.9%)	1/3 (33.3%)
	Synchronous bowel tumors^b^		84/240 (35.0%)	
SRI	Mesenteric metastases		64/88 (72.7%)	
	Peritoneal metastases		2/14 (14.3%)	
	Liver metastases^a^		13/17 (76.5%)	
	Synchronous bowel tumors^b^		84/135 (62.2%)	
Overall preoperative assessment	Correct T‐stage		23/100 (23.0%)	4/12 (33.3%)
	Correct N‐stage		57/100 (57.0%)	2/12 (16.7%)
	Correct M‐stage		85/100 (85.0%)	11/12 (91.7%)
	Correct tumor entity	118/187 (63.1%)	100/108 (92.6%)	12/24 (50.0%)
Intraoperative palpation	Mesenteric metastases		76/97 (78.4%)	10/17 (58.8%)
	Synchronous bowel tumors^b^		292/307 (95.1%)	

*Note:* Results of preoperative investigations compared to final histopathological results if not otherwise specified. Number of patients in denominator if not otherwise specified.

^a^ Held against other perioperative liver imaging or intraoperative findings.

^b^ Number of luminal bowel tumors, histopathological tumor number in denominator.

SRI was positive in 82/91 (90%) siNET and 6/28 (21%) non‐siNET patients. All SRI negative siNET were grade 1 tumors. Sensitivity for siNET disease was 87% for ^99m^Tc‐Tectrotyde scintigraphy (Tc‐scint) and 90% for ^68^Ga‐DOTATOC PET‐CT (Ga‐PET, Table 4). SRI identified an N+ status in 64/88 (73%), with no significant difference between Tc‐scint (24/36; 67%) and Ga‐PET (42/55; 76%; *p* = 0.34). Pathologists found 285 bowel tumors in the 91 siNET patients who had an SRI. In 17 (19%) of these patients, no bowel tumor was detected on SRI. Endoscopic biopsy (*n* = 11), SRI‐positive mesenteries (*n* = 8) and suggestive CT findings (*n* = 10) still ensured that all these patients had a correct tentative preoperative tentative diagnosis. In 85 patients with complete tumor number data, SRI missed 51/135 (38%) of histopathologically found tumors. Tc‐scint underestimated synchronous siNET numbers in 18/39 (46%) patients compared to 14/57 (25%) for Ga‐PET (*p* = 0.05). At the tumor level, Tc‐scint missed 27/57 (47%) tumors versus 32/91 (35%) for Ga‐PET (*p* = 0.17).

**TABLE 4:** Somatostatin receptor imaging (SRI) of small intestinal neuroendocrine tumors (siNET).

Outcome measure	Tc‐scint (*n = 39)*	Ga‐PET (*n = 57)*
Sensitivity for siNET disease	34/39 (87.1%)	51/57 (89.5%)
Number of patients with underestimated tumor number	18/39 (46.2%)	14/57 (24.6%)
Missed synchronous siNET^a^	27/57 (47.4%)	32/91 (35.2%)
Mesenteric metastases (N+) sensitivity	24/36 (66.7%)	42/55 (76.4%)
Liver metastases sensitivity^b^	6/8	7/9
Peritoneal metastases sensitivity	0/4	2/10

*Note:* ^99m^Tc‐Tectrotyde scintigraphy (Tc‐scint) and ^68^Ga‐DOTATOC PET‐CT (Ga‐PET) results. During the study period, T‐scint was replaced by Ga‐PET; no patient had T‐scint alone after Jan 2020, two patients had Ga‐PET as their sole SRI before Jan 2020.

^a^ Histopathological tumor number in denominator.

^b^ Liver metastases in perioperative magnetic resonance imaging (MRI) in four patients with negative livers in SRI.

Intraoperative palpation failed to identify 15/307 (5%) histopathologically verified luminal siNET (in 100 patients with complete data). Intraoperatively suspected N+ mesenteries were verified by histopathology in 76/97 (78%) siNET patients. Solitary terminal siNET were more often SRI negative than more proximal tumors (17% vs. 6%, *p* < 0.001). The characteristics of terminal ileum siNET are presented in Figure 3. The main findings are summarized in Table 5.

**FIGURE 3:**
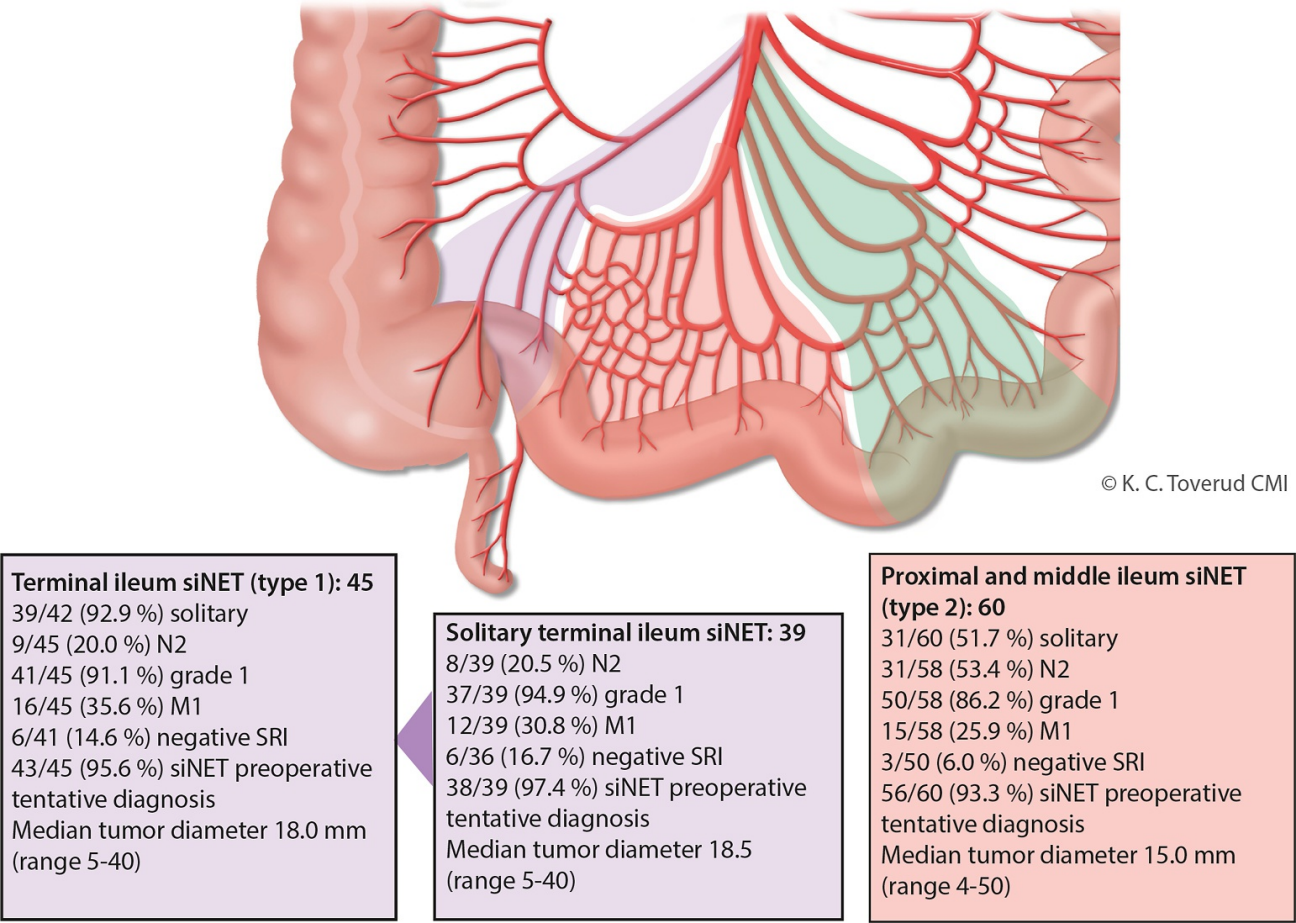
Subgroups of siNET. Terminal (type1) compared to middle/upper ileum (type 2a + 2b) siNET were more often solitary and less often N2. Terminal siNET were less often SRI positive but within reach of ileocoloscopy. siNET: small intestine neuroendocrine tumor, SRI: somatostatin receptor imaging, N2: ≥ 12 mesenteric metastases and/or mesenteric mass diameter > 2 cm, M1: distant metastases present.

**TABLE 5:** Summary of main findings. Small bowel tumor types and their main features.

Entity	Hallmarks
Small intestinal neuroendocrine tumor (siNET) (*n* = 108)	All in the ileum Distal tumors more often solitary Distal tumors less extensive mesenteric disease Multiple bowel tumors (2–61 tumors) in 31% Tumor multiplicity associated with distant metastases Sensitivity for synchronous bowel tumors in CT 35%, in somatostatin receptor imaging 62%, by intraoperative palpation 95%^a^ Sensitivity for mesenteric metastases (N+ stage) in CT 72%, in somatostatin receptor imaging 73%, by intraoperative palpation 78%^a^ Sensitivity for carcinomatosis in CT 13%, in somatostatin receptor imaging 14%^b^
Small intestinal carcinoma (n = 24)	Duodenojejunal junction location in 42%, Proximal cancers more often nodal dissemination but less often distant metastases Most often symptomatic and solitary tumors
Gastrointestinal stromal tumor (GIST) (n = 13)	All solitary tumors
Lymphoma (n = 8)	All multiple mesenteric tumors
Malignant melanoma metastasis (n = 7)	Small bowel obstruction as melanoma disease debut in 6/7
Desmoid tumor (n = 4)	All large mesenteric tumor deposits
Benign lesion (n = 13)	Rarely mistaken for cancer and mostly sorted out in diagnostic work‐up. Ectopic pancreatic tissue (*n* = 1), leiomyoma (*n* = 1), lipoma (*n* = 1), endometriosis (*n* = 1), adenomatous polyps (*n* = 2), fibrous polyp (*n* = 1) inflammatory granuloma (*n* = 1), mesenteric sclerosis (*n* = 1), cystic Gorlin's syndrome bowel lesion (*n* = 1), no tumor found (*n* = 3) Indication for surgery suspicion of malignancy in CT (*n* = 8) or in PET scans (*n* = 4), or obstructive invagination (*n* = 1)

*Note:* The cohort also includes one case of primary leiomyosarcoma and nine metastases from other organs: ovary (*n* = 1), colon (*n* = 2), tongue (*n* = 1), uterus (*n* = 1), lung (*n* = 3) and breast (*n* = 1).

^a^ Compared to histopathology.

^b^ Compared to intraoperative findings with histopathological biopsies.

## 4 Discussion

This 10‐year single‐center experience demonstrates the complexity of preoperative work‐up of small bowel cancer and its treatment implications. The main findings are the substantial number of preoperatively undetected tumor manifestations and the localization and dissemination pattern of tumors, with subsets of disease. Surgeons must be prepared to adapt their planned strategy based on intraoperative findings.

Although the small bowel cancer work‐up in this study incorporated a broad spectrum of specialized investigations, the preoperative tentative diagnosis was in accordance with the final histopathological diagnosis in only 63% of patients. Even if the precise diagnosis might be missing, CT scans correctly identified the presence of malignant disease in 91%. CT sensitivity for mesenteric metastases was low, for carcinomatosis even lower, and SRI did not perform any better. However, SRI demonstrated its known [34] efficacy in establishing the diagnosis of well‐differentiated siNET with a sensitivity of 90%, Tc‐scintigraphy performing almost equal to Ga‐PET, which is with less difference than expected from known PET and scintigraphy comparisons [11].

Despite the many diagnostic innovations in recent years, the study revealed persistent shortcomings in tumor identification. A siNET multiplicity of 31% is a little less than previously reported [35]. CT scans failed to identify 156 synchronous tumors, and 51 tumors were not detected in SRI. Bowel tumors were missed by SRI in 30 (33%) patients. Supporting Information S1: Figure 1 illustrates a case of 12 siNET of which only four were seen preoperatively. SiNET multiplicity was associated with distant metastases. Published results are equivocal in this matter [36, 37].

The present study supports the observation that there has been a change in Western countries toward siNET being the most prevalent small bowel cancer type. The rising siNET incidence has been attributed to an increased use of diagnostic imaging [38]. Lymph node metastases (N+) were found in 95% of siNET patients and distant metastases (M+) in 33%, M+ stage being less frequent than published [39, 40]. Lymph node metastases were found in 74% of adenocarcinoma patients, which is more frequent than previously reported [41, 42]. Intraoperative examination improved the staging precision for both adenocarcinoma and siNET. Duodenojejunal carcinomas were less frequent M+ and showed a tendency to be more often N+ than more distal carcinomas. This finding should be interpreted with caution due to the small sample size. More nodal involvement in duodenal adenocarcinomas than in jejunoileal cancers has been previously described [43]. The occurrence of several lymphomas, desmoids, and benign lesions in this material underscores the lack of sensitivity to precisely identify these lesions that usually don't need to be removed by surgery. Modern lymphoma and desmoid tumor treatment include surgery only when obstruction or perforation is imminent [44, 45].

A novel approach to define tumor localization was used, based on feeding arteries and location of tumors and mesenteric metastases. Adenocarcinomas were found in all parts of the small bowel and dominated in the duodenojejunal sector. siNET were exclusively found in the ileal sectors, which is a much more pronounced distal affinity than previously published [46, 47]. The reasons behind this has been proposed to be of chemical‐physiological [48], anatomical [49], embryological [50] or genetic [51] character. In contrast to tumors in more proximal parts of the ileum, the subgroup of siNET in the terminal ileum are predominantly solitary with a lower risk of troublesome large mesenteric masses. Subgroup identification could enable more individualized surgery. Especially for siNET patients, who have a good long‐term survival [52], it is crucial to balance potential considerable symptom burden caused by remaining or recurrent disease [53] against that inflicted by extensive interventions [20].

All gathered information is of crucial importance to the surgeon's decisions, both for bowel resection levels, vessel ligation and extent of lymphadenectomy. The number of mesenteric metastases not detected pre‐ or intraoperatively supports standardized and radical lymphadenectomy. Minimally invasive procedures have demonstrated benefits, in postoperative recovery, complication rates and incisional hernia frequencies [54]. Laparoscopy is described in siNET surgery, with high conversion rates [55]. Our findings suggest that in small bowel cancer surgery, where siNET is the most important differential diagnosis, a totally minimally invasive procedure would risk leaving tumor tissue behind. Laparoscopic or robotic procedures are of value in abdominal cavity exploration, mobilization, dissection and central lymphadenectomy, but operations should include inspection of the entire peritoneal cavity and a mini laparotomy with manual palpation of the small bowel and its mesentery. The need of longer [46] or multiple [35] bowel resections has been demonstrated in multiple siNET. The affected bowel lengths in the present study were limited—even though cases of excessive multiplicity were included. As siNET were not found in upper bowel parts, smaller incisions seem to be sufficient for exteriorization. The frequently negative SRI in the terminal ileum siNET can to some extent be remedied by ileocoloscopic accessibility. Endoscopy has a place in the work‐up of tumors at the duodenojejunal angle and in the terminal ileum, where SRI of siNET was frequently negative. However, in our opinion, enteroscopies and biopsies can be omitted when SRI is positive. A proposed work‐up is presented in Figure 4.

**FIGURE 4:**
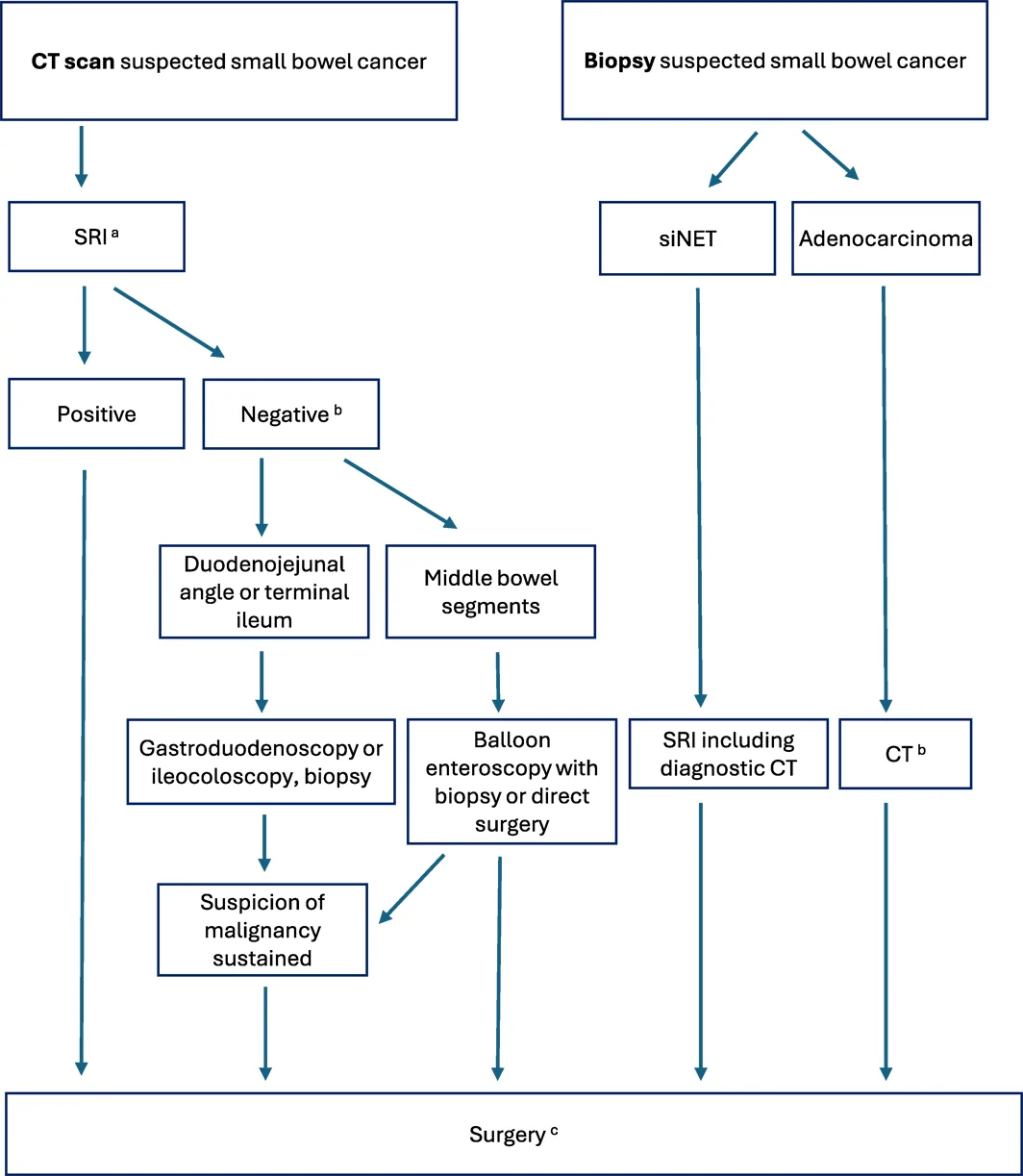
Proposed work‐up of small bowel cancer. CT = computed tomography; SRI = somatostatin receptor imaging; siNET = small intestinal neuroendocrine tumors. ^a^ Cases with typical CT features of desmoids or lymphomas are investigated further by transabdominal biopsies and cases with typical CT features of gastrointestinal stromal tumors (GIST) could proceed directly to surgery ^b^ A thoracic CT scan is included ^c^ Minimally invasive techniques are supplemented by a mini laparotomy for manual palpation of bowel and mesentery whenever siNET is a possibility. Treatment choices are supported by multi‐disciplinary boards and patient shared decision making. Surgery is usually not advised in cases of disseminated non‐curable adenocarcinoma disease. For siNET, resection of bowel tumors and mesenteric disease is often advised even in cases with distant metastatic disease.

The strength of this material is the broad and detailed analysis of an up‐to‐date, complete and consecutive cohort of patients operated at the same public hospital treated by the same clinicians. The weakness is the retrospective design—and that low absolute numbers challenge statistical analyses. Conclusions should be drawn with caution and are merely hypothesis‐generating. There are controversies regarding the sectorizing of the small bowel. There is no established consensus on where to define the border between the jejunum and ileum; both a gradual transition [56] and a defined boundary at the level of the ileocolic artery (ICA) origin [57] is advocated. Anatomical variations may, albeit rarely, introduce definitional challenges, as the ileocolic artery (ICA) may be absent or share a common stem with the middle colic artery (MCA) [58, 59]. It is also of note that a short distance between ICA and MCA has been shown when ICA crosses anterior to the superior mesenteric vein [60]. However, all tumors in this material could be sectorized.

## 5 Conclusion

Modern diagnostic work‐up identifies malignant small bowel disease with few false positives but remains unreliable for accurate tumor characterization. Tumor multiplicity and dissemination are difficult to predict. Using a novel five‐sector classification, adenocarcinomas were found throughout the small bowel, whereas siNET were confined to the ileum. We recommend an adaptive surgical approach including minimally invasive surgery, peritoneal cavity inspection and a mini laparotomy for palpation of bowel and mesentery. The frequent siNET multiplicity is associated with distant metastases. Distinct tumor subgroups may exist at the proximal and distal extremes, warranting further study.

## Author Contributions

**Erik Kjaestad:** conceptualization, investigation, writing – original draft, methodology, validation, visualization, writing – review and editing, software, formal analysis, project administration, data curation, resources. **Johannes Kurt Schultz:** conceptualization, investigation, writing – original draft, methodology, validation, visualization, writing – review and editing, software, project administration, supervision, resources. **Arne Olav Bakka:** conceptualization, investigation, writing – original draft, writing – review and editing, methodology, project administration, supervision. **Espen Thiis‐Evensen:** conceptualization, investigation, writing – original draft, writing – review and editing, methodology, project administration, supervision. **Anita Sveen:** conceptualization, investigation, writing – original draft, writing – review and editing, supervision, project administration.

## Funding

The authors have nothing to report.

## Ethics Statement

The study was approved by the southeast Norwegian regional ethics committee (ID number753582).

## Conflicts of Interest

The authors declare no conflicts of interest.

## Supporting information


Supporting Information S1

## Data Availability

The data that support the findings of this study are available on request from the corresponding author. The data are not publicly available due to privacy or ethical restrictions.
