# Supplementary material for: Small Bowel Cancer: Implications of Preoperative Work‐Up on Surgical Strategy
Source: World J Surg. 2026 Sep 3;50(10):3008–19. doi: 10.1002/wjs.70547 (PMC13662508; doi:10.1002/wjs.70547)
Supplement: Supplementary file 1 [file WJS-50-3008-s001.docx]

Supplementary

**Supplementary figure 1. Tumor multiplicity** **hard to detect.** From top left: CT and ^68^Ga-DOTATOC-PET, operative and specimen photos. Of twelve luminal siNET only four were visualized preoperatively, alongside a mesenteric mass. Arrows point to a siNET and its mesenteric metastasis. The superior mesenteric vessels are ligated and lymphadenectomy performed (type 2a)

| **Histopathology** | | **siNET (n=108)** | | **Carcinoma (n=24)** | |
| --- | --- | --- | --- | --- | --- |
| **Differentiation** | | Grade 1  (Ki-67 <3%) | 95 (88.0%) | Adenocarcinoma high differentiation | 5 (20.8%) |
|  |  |  |  | Adenocarcinoma moderate differentiation | 4 (16.7%) |
|  |  | Grade 2  (Ki-67 3-20%) | 12 (11.1%) | Adenocarcinoma poor differentiation | 9 (37.5%) |
|  |  |  |  | Mucinous adenocarcinoma | 3 (12.5%) |
|  |  | Grade 3  (Ki-67>20%) | 1 (0.9%) | Adenocarcinoma not specified | 1 (4.2%) |
|  |  |  |  | Undifferentiated carcinoma | 1 (4.2%) |
|  |  |  |  | Adenosquamous carcinoma | 1 (4.2%) |
| **T stage** | T0 | 3 (2.8%) | | 0 | |
|  | T1 | 10 (9.3%) | | 0 | |
|  | T2 | 18 (16.7%) | | 1 (4.2%) | |
|  | T3 | 24 (22.2%) | | 6 (25.0%) | |
|  | T4 | 49 (45.4%) | | 17 (70.8%) | |
|  | Tx | 3 (2.8%) | |  | |
| **N stage** | N0 | 5 (4.6%) | | 6 (25.0%) | |
|  | N1 | 56 (51.9%) | | 12 (50.0%) | |
|  | N2 | 42 (38.9%) | | 5 (20.8%) | |
|  | Nx | 5 (4.6%) | | 1 (4.2%) | |
| **M stage** | M0 | 72 (66.7%) | | 14 (58.3%) | |
|  | M1 | 36 (33.3%) | | 10 (41.7%) | |
| **Overall stage** | 1 | 2 (1.9%) | | 0 | |
|  | 2 | 3 (2.8%) | | 3 (12.5%) | |
|  | 3 | 65 (60.2%) | | 11 (45.8%) | |
|  | 4 | 36 (33.3%) | | 10 (41.7%) | |
|  | x | 2 (1.9%) | |  | |

**Supplementary table 1. Histopathology** of the small intestinal neuroendocrine tumors (siNET) and the primary carcinomas. For one siNET, Ki67 analysis failed and grade 1 was established by morphologic features. T0; no primary found. Tx, Nx: no assessment.
